# A phase 1 trial of the safety, tolerability and biological effects of intravenous Enadenotucirev, a novel oncolytic virus, in combination with chemoradiotherapy in locally advanced rectal cancer (CEDAR)

**DOI:** 10.1186/s13014-020-01593-5

**Published:** 2020-06-12

**Authors:** Séan M. O’Cathail, Steven Davis, Jane Holmes, Richard Brown, Kerry Fisher, Leonard Seymour, Richard Adams, James Good, David Sebag-Montefiore, Tim Maughan, Maria A. Hawkins

**Affiliations:** 1 Oxford Institute of Radiation Oncology, University of Oxford, Oxford, OX3 7LE UK; 2 Department of Oncology, University of Oxford, Oxford, OX3 7LE UK; 3 Centre for Statistical Medicine, University of Oxford, Oxford, OX3 7LE UK; 4 PsiOxus Therapeutics, Barton Lane, Abingdon, OX14 3YS UK; 5 Velindre Cancer Centre, Cardiff, CF14 2TL UK; 6 Queen Elizabeth Hospital Birmingham, Edgbaston, Birmingham, B15 2GW UK; 7 Department of Clinical Oncology, University of Leeds, Leeds Cancer UK Centre, Leeds, UK; 8 Department of Physics and Biomedical Engineering, University College London, Oxford, UK

**Keywords:** Radiation, Phase I, Rectal, Cancer, Oncolytic virus, Immuno-oncology

## Abstract

**Background:**

Chemoradiotherapy remains the standard of care for locally advanced rectal cancer. Efforts to intensify treatment and increase response rates have yet to yield practice changing results due to increased toxicity and/or absence of increased radiosensitization. Enadenotucirev (EnAd) is a tumour selective, oncolytic adenovirus which can be given intravenously. Pre-clinical evidence of synergy with radiation warrants further clinical testing and assessment of safety with radiation.

**Methods:**

Eligibility include histology confirmed locally advanced rectal cancer that require chemoradiation. The trial will use a Time-to-Event Continual Reassessment Model-based (TiTE-CRM) approach using toxicity and efficacy as co-primary endpoints to recommend the optimal dose and treatment schedule 30 patients will be recruited. Secondary endpoints include pathological complete response the neoadjuvant rectal score. A translational program will be based on a mandatory biopsy during the second week of treatment for ‘proof-of-concept’ and exploration of mechanism. The trial opened to recruitment in July 2019, at an expected rate of 1 per month for up to 4 years.

**Discussion:**

Chemoradiation with Enadenotucirev as a radiosensitiser in locally Advanced Rectal cancer (CEDAR) is a prospective multicentre study testing a new paradigm in radiosensitization in rectal cancer. The unique ability of EnAd to selectively infect tumour cells following intravenous delivery is an exciting opportunity with a clear translational goal. The novel statistical design will make efficient use of both toxicity and efficacy data to inform subsequent studies.

**Trial registration:**

ClinicalTrial.gov, NCT03916510. Registered 16th April 2019.

## Background

Locally advanced rectal cancer (LARC) is defined by the presence of T3/4 disease and/or nodal involvement in the absence of distal metastatic disease. For many years the standard of care has been a multimodal approach incorporating neoadjuvant long-course chemoradiotherapy (CRT) with concurrent fluoropyrimidine, followed by total mesorectal excision surgery [1]. With this approach, local recurrence rates have fallen from 30 to 45% to < 10%. Nevertheless, responses to treatment remain heterogeneous with up to 14% responding completely, 20% with little or no regression and the remainder displaying a spectrum between these two extremes [2]. Higher response rates are associated with improved outcomes [3] and innovative combinations with standard of care radiation are an area of intense interest [4, 5]. In particular, combining radiotherapy with immunotherapy is emerging as a further opportunity to improve therapeutic results and is a seen as a priority for research. Using a novel oncolytic adenovirus, Enadenotucirev (EnAd), we aim to elicit these benefits in rectal cancer.

Oncolytic viruses are novel anticancer agents that target, selectively replicate in, and kill cancer cells, spreading through the tumour microenvironment, generating local and systemic immune response both to themselves as well as the dying tumour. One principal advantage of oncolytic therapy is that the drug (the virus) replicates only in malignant cells meaning that the concentration of drug is amplified at the site of pathology so that it is higher in the tumour than in healthy tissue; completely different to standard drug pharmacokinetics. Virus particles can also spread from cell to cell within a tumour nodule until they reach non-permissive normal tissues, in principle targeting all viable tumour cells they encounter [6, 7].

### Rationale for the trial

There is a wealth of evidence to support the rationale for combining this class of agent with radiation and has recently been well reviewed [8]. Radiotherapy can modulate the expression of a large number of cellular genes involved in cell cycle checkpoints, cellular stress, DNA repair and apoptosis [9]. Adenoviruses have developed a range of interactions with cellular DNA damage repair proteins to allow successful viral replication. This has implications for the initiation of a number of DNA repair pathways activated in response to radiation-induced damage, in particular, all adenoviral serotypes appear to target Non Homologous End Joining (NHEJ) repair [10]. The hypothesis that oncolytic adenovirus infection would work synergistically with radiotherapy has been tested by a number of groups. The combination of CG7870 adenovirus with radiation resulted in a synergistic increase in cell killing, both in-vitro and in-vivo in the LNCaP xenograft model, than either agent alone [11]. Three Ad5-based vectors combined with radiation have been studied in A549 lung cancer cells [12]. In-vivo and in-vitro tumour cell kill was increased with the combination approach. Similar findings have been noted with a variety of different adenoviral vectors in other cell types including ovarian cancer cell lines [13] and glioma xenografts [14]. Importantly the effect of radiosensitization does not appear to extend to normal tissues. The combination of Ad5/CMV/p53 synergistically radiosensitized two non-small cell lung cancer cell lines (A549 and H322) in-vitro and in xenograft models, in a synergistic fashion, but showed no increased radiosensitization effect on normal lung fibroblasts [15].

Clinical experience with virus/radiation combinations has been limited to local (most commonly intratumoural) administration. This mode of delivery facilitates direct infection ensuring correct dosing and avoids rapid hepatic uptake seen with systemic delivery [16]. The drawback was only tumour types that can be accessed with a needle, such as skin, head and neck cancers, prostate cancers, were considered suitable for clinical trials. Nevertheless, the results of these studies provide useful mechanistic indications as well as guiding assessment of toxicity. Early phase evidence supports the hypothesis that oncolytic adenovirus’ act to enhance radiation damage across a range of tumour types including prostate [17–20], head and neck SCC [21], pancreas [22], lung [23] and CNS [24], with largely acceptable side effects and toxicity profile [19, 25–28].

#### Enadenotucirev

Enadenotucirev is a group B oncolytic adenovirus under development for the systemic treatment of metastatic or advanced epithelial tumours. A chimeric adenovirus type 11p (Ad11p)/adenovirus type 3 (Ad3) virus, it was developed through a process of bio-selection where pools of adenovirus serotypes are passaged on human tumor cell lines to invite recombination of potent serotypes, in a non-prejudicial way. Thus the human tumor directs the evolution of select, highly potent adenovirus serotypes. EnAd was derived from a pool of seven different adenoviruses serotypes utilizing human HT-29 colorectal cancer (CRC) cells [29]. As a consequence of this process, EnAd shows selective and potent toxicity in human carcinoma cells with limited toxicity to normal (non-cancerous) human cells. Other than humans, there is no known permissive species for EnAd.

While the overall understanding of the mechanism of action of EnAd in humans is evolving, it is well established from both non-clinical and clinical studies that the mechanism of anti-cancer efficacy of oncolytic viruses elicit two primary effects; direct infection and lysis of tumour cells and stimulated immune responses via increased release of tumour-associated antigens and immune-inflammatory activation signals. The direct oncolytic efficacy of EnAd has been demonstrated in a number of non-clinical studies with human tumour cells in-vitro and in-vivo [29, 30]*.* Replication of the enadenotucirev virus in carcinoma cells resulted in direct necrolytic killing of carcinoma cells by a non-apoptotic, immunogenic cell death mechanism which would be expected to trigger immune cells.

Two clinical studies of enadenotucirev given as a monotherapy are important. The mechanism of action study (ColoAd1–1002) established that intravenous delivery of EnAd was as efficient as intratumoral delivery in colorectal cancer [31], making EnAd unique. The EVOLVE (Evaluating Oncolytic Vaccine Efficacy) study was a dose escalation and dosing schedule evaluation in metastatic epithelial solid tumours which has established the monotherapy maximum tolerated dose (MTD) [32]. Common adverse events associated with EnAd include asthenia, flu like symptoms, nausea, vomiting,, pyrexia and fatigue.

#### Aims of the trial

Our hypothesis is that enadenotucirev will, selectively, downregulate DNA repair pathways in rectal cancer cells, making them more susceptible to DNA damage already incurred. Enadenotucirev also has the potential to induce an immunogenic cell death in malignant cells adding a complimentary, cytotoxic mechanism of action. Enadenotucirev would address the combined requirements as therapy could act as both a local sensitizer (DDR inhibitor/ direct tumour kill) and systemic (immune response) agent. The aim of the trial is to find the treatment schedule that has the optimal response-toxicity trade-off, with no more than 30% probability of a DLT. This is based on a historical G3+ adverse event rate for CRT of approximately 30% [33, 34]. Modern radiotherapy techniques means toxicity is expected to be lower from CRT and recent studies with novel radiosensitizers such as oxaliplatin reported G3/G4 toxicity in the order of 25% [2].

## Design/methods

**Fig. 1:**
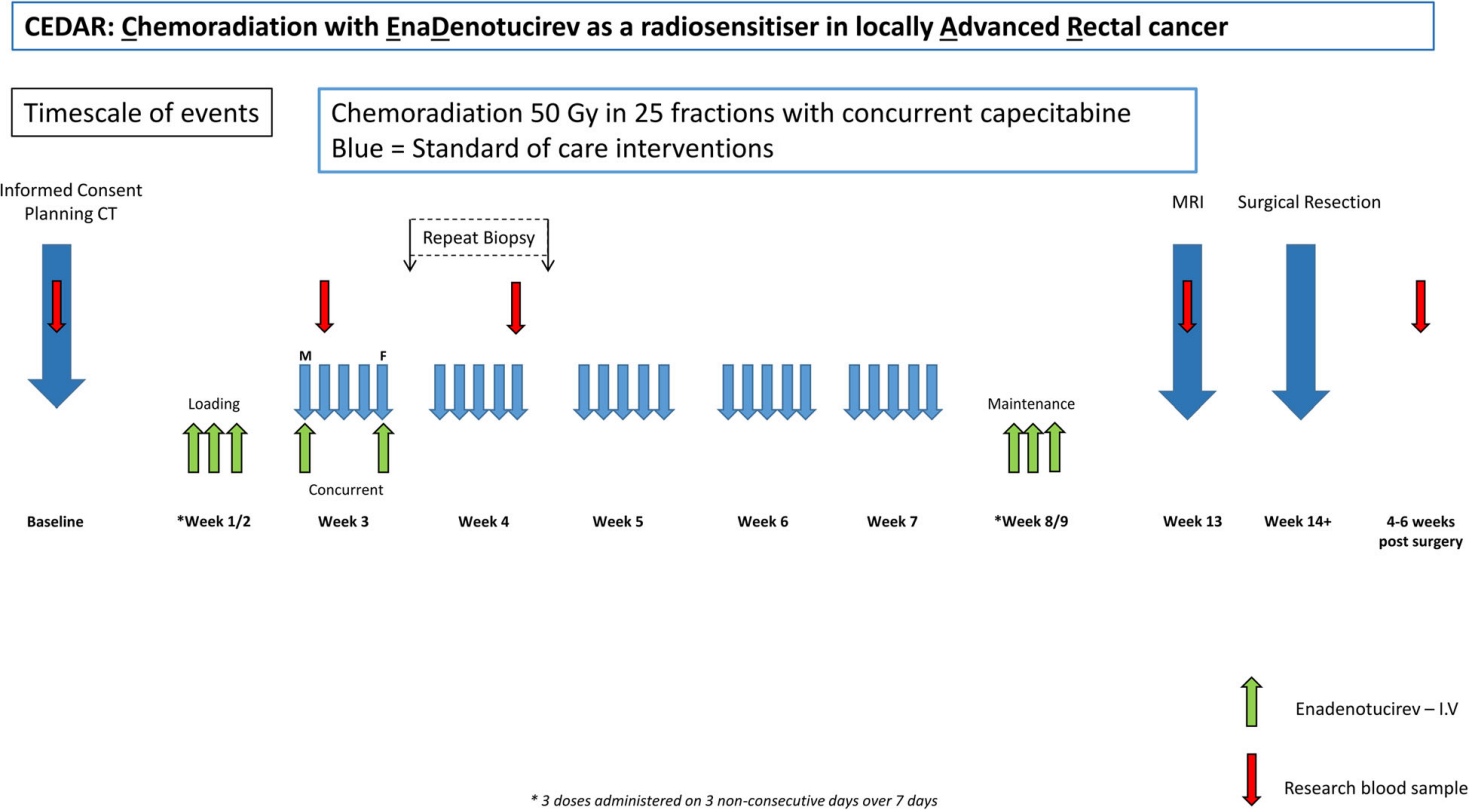
Overview of the study schema indicating timelines and interventions. [Blue = standard of care interventions; Green = viral particle administrations; Red = investigational blood sample retrieval]

CEDAR is a dual endpoint, dose escalation phase I trial using a time to event continual reassessment method (TiTE CRM). Response and toxicity endpoints will be combined in dose escalation models to identify the optimal dose schedule. We will recruit a maximum of 30 patients. Four centres will recruit to the study. Dose escalation will be achieved by first increasing the frequency of administration of EnAd followed by increasing the viral particle dose of EnAd as detailed in the trial flow chart (Fig. 1). These dose schedules are considered ordered with increasing toxicity expected from one dose schedule to the next.

### Objectives

To determine the optimal dose and frequency of enadenotucirev that can be administered with chemoradiation for rectal cancer.

Primary Endpoints

- Dose limiting toxicity
- MRI tumour regression grade

Secondary Endpoints

- Ability to deliver enadenotucirev concurrently with chemoradiation
  - Assessment of treatment tolerance as measured by the proportion of patients completing at least 80% of the intended Capecitabine dose and at least 20 fractions of radiotherapy by the end of week 9
- To measure local response rate to combined therapy compared to pre-treatment status
  - Pathological complete response rate
  - Neoadjuvant rectal (NAR) score [35]

Exploratory Endpoints

- To identify ‘proof of concept’ that enadenotucirev replicates in the tumour during chemoradiotherapy
  - IHC staining of hexon protein coat in tumour cells from ‘on-treat’ biopsy performed on week 2 of radiotherapy

### Study population

#### Inclusion criteria

A patient will be eligible for inclusion in this trial if all of the following criteria apply.

- Histologically confirmed invasive adenocarcinoma of the rectum.
- Locally advance colorectal cancer as defined by pelvic MRI with a threatened circumferential resection margin (cT3mrf + ve), or inclusion of an adjacent organ, or low tumours at/below the level of the levators or enlarged pelvic side wall nodes or selected by the multidisciplinary team MDT for treatment with neoadjuvant (chemo)radiotherapy, regardless of TNM classification
- Patients with oligometastatic disease suitable for radical treatment are permitted provided that the site specific MDT deems them suitable for chemoradiation
- Male or female, Age ≥ 18 years.
- ECOG performance score of 0–1
- The patient is willing and able to comply with the protocol scheduled biopsy, follow-up visits and examinations for the duration of the trial.
- Written (signed and dated) informed consent.
- Adequate renal function demonstrated by:
  - Creatinine ≤1.5 mg/dL and estimated glomerular filtration rate (eGFR) ≥60 mL/min/1.73 m2 (or measured creatinine clearance ≥60 mL/min)
  - Urine dipstick for proteinuria at screening and baseline negative or trace. Patients may be included with results of 1+ if they have a spot urinary albumin creatinine ratio (ACR) of either:
  - ≤3 mg/mmol or
  - > 3 mg- < 70 mg/mmol with a 24 h urinary protein < 0.2 g/24 h and
  - Serum complement components C3 and C4 within the normal range

Exclusion criteria

- Pregnant or breast-feeding women, or women of childbearing potential unless effective methods of contraception are used.
- Past medical history:
  - Known history or evidence of significant immunodeficiency due to underlying illness and/or medication (e.g. systemic corticosteroids, or other immunosuppressive medications including cyclosporine, azathioprine, interferons in the 4 weeks before the first dose of trial treatment)
  - Splenectomy
  - Prior allogeneic or autologous bone marrow or organ transplantation
  - Patients with a history of, or active, known or suspected auto-immune disease or a syndrome that requires systemic or immunosuppressive agents; patients with vitiligo, type I diabetes mellitus, residual hypothyroidism due to autoimmune disease only requiring hormone replacement, psoriasis not requiring systemic treatment or conditions not expected to recur in the absence of an external trigger are permitted to enrol
  - History of idiopathic pulmonary fibrosis, drug-induced pneumonitis, or evidence of active pneumonia or pneumonitis on computed tomography scan
  - Active viral disease or known positive serology for HIV, hepatitis B or hepatitis C
  - Active infections requiring antibiotics, physician monitoring, or recurrent fevers > 38.0 °C associated with a clinical diagnosis of active infection
  - Prior pelvic radiotherapy
  - Any other active malignancy, with the exception of adequately treated cone-biopsied in situ carcinoma of the cervix uteri and non-melanoma skin lesions.
  - Uncontrolled cardiorespiratory comorbidity (e.g. inadequately controlled angina or myocardial infarction in the last 6 months)
  - Major disturbance in bowel function (e.g. severe incontinence, Crohn’s disease, > 6 loperamide/day)
- Use of the following antiviral agents: ribavirin, adefovir, lamivudine or cidofovir within 7 days prior to the first dose of trial treatment; or pegylated interferon in the 14 days before the first dose of trial treatment
- Treatment with any other investigational agent, or participation in another interventional clinical trial within 28 days prior to enrolment. Observational studies are allowed
- Warfarin that cannot be discontinued at least 7 days prior to starting treatment
- Known dihydropyrimidine dehydrogenase (DPYD) deficiency
- Prior chemotherapy is allowed as long as > 28 days since the last administration and any toxicity has resolved to NCI CTCAE grade 1 or less
- Other psychological, social or medical condition, physical examination finding or a laboratory abnormality that the Investigator considers would make the patient a poor trial candidate or could interfere with protocol compliance or the interpretation of trial results.

#### Statistical design

The trial will use a Time-to-Event Continual Reassessment Model-based (TiTE-CRM) approach using toxicity and efficacy primary endpoints to recommend the dose and treatment schedule for future patients. MRI regression was chosen as the primary response endpoint, as opposed to a pathological metric, so that response could be assessed in all patients of a small cohort even in cases of organ preservation. In using response alongside toxicity to inform the selection of the best dose, the trial does not assume that an increase in dose causes an increase in efficacy. This means that if the dose with the best response rate is below the maximum tolerable dose then it may be selected as the optimal dose. It also means that marginal gains in efficacy which do not justify large increases in toxicity can be avoided. The models for the two endpoints are Bayesian logistic models with weakly informative priors calibrated to ensure the model provides sensible recommendations during the early part of the study. Both models will use all available information for every dose decision [36, 37]. Patients who have not reached the efficacy time point yet will not provide any information to the model. Patients who have reached this time point but did not have the evaluation, withdrew or died prior to evaluation will be treated as non-responders. A further restriction on escalation is that we will only escalate to an untried dose if at least 2 patients have been given the dose below and followed for at least 8 weeks.

The first 2 patients have been recruited and followed until the end of the DLT window. From now on patients will be recruited continuously and toxicity and response models will be fitted every time a new patient is registered to decide which dose they will receive. We used simulations to justify a sample size of 30.

#### Approvals

The trial is sponsored by the University of Oxford and was approved by the South Central - Oxford B Research Ethics Committee (REC Reference: 18/SC/0583). The study is part funded by Cancer Research UK, PsiOxus Therapeutics and adopted into the CT-RAD/NCRI portfolio.

### Interventions

#### Chemoradiotherapy

Patients registered for the trial will receive standard chemoradiation treatment which consists of Capecitabine 900 mg/m2 orally twice a day in equal doses (Mon-Fri) on the days of radiotherapy for 25 daily treatments [38]. The radiotherapy protocol mandates the use of intravenous contrast CT simulation with minimum 3 mm CT slices. Patients are immobilised supine with customised pelvic immobilization equipment, with a comfortably full bladder. 50Gy in 25 fractions will be delivered to the primary tumour (GTVp) and macroscopically involved lymph nodes (GTVn), as a simultaneous integrated boost, and 45Gy in 25 fractions to the pelvis/mesorectal nodes and elective pelvic lymph nodes at risk (CTV), prescribed according to recommendations by the International Commission on Radiation Units and Measurements (ICRU-50/62), to be delivered Monday to Friday as an intensity modulated radiotherapy planned single-phase treatment. An adapted atlas is provided to aid with radiotherapy planning each case is reviewed to ensure consistency of radiotherapy delineation and radiotherapy dose delivery. Patients are reviewed weekly during RT (as per standard of care) and prior to each administration of enadenotucirev.

#### Enadenotucirev

All participants will receive 3 x loading doses intravenously in weeks 1–2, prior to initiation of chemoradiotherapy. Loading doses, and maintenance doses if assigned, should be given on 3 non-consecutive days over a 7-day period (e.g. Mon/Wed/Fri or Fri/Mon/Wed or other convenient schedule). Further doses of enadenotucirev after the 3 loading doses are dependent on the dose schedule assigned, as per Table 1. As premedication, all patients will receive 100 mg IV hydrocortisone and 1 g oral paracetamol one hour prior to EnAd and again three hours after EnAd.

**Table 1:** Dose levels used for dose escalation. Two separate viral particle doses are used with escalation achieved by increasing the frequency of administration. Administrations of the virus are highlighted by the green arrows in Fig. 1

Dose schedule	Loading (Pre CRT) (3 doses given on 3 non-consecutive days over a 7 day period)	Concurrent		Maintenance (post CRT)(3 doses given on 3 non-consecutive days over a 7 day period)
		Week 1 Day 1 CRT (week 3 day 1)	Week 1 Day 5 CRT (week 3 day 5)	
**1 (start level)**	**1 × 10**^**12**^**vp**			
**2**	**1 × 10**^**12**^**vp**			**1 × 10**^**12**^**vp**
**3**	**1 × 10**^**12**^**vp**	**1 × 10**^**12**^**vp**	**1 × 10**^**12**^**vp**	**1 × 10**^**12**^**vp**
**4**	**3 × 10**^**12**^**vp**			
**5**	**3 × 10**^**12**^**vp**			**3 × 10**^**12**^**vp**
**6**	**3 × 10**^**12**^**vp**	**3 × 10**^**12**^**vp**	**3 × 10**^**12**^**vp**	**3 × 10**^**12**^**vp**

#### Biopsy on treatment

The trial mandates a biopsy in the second week of CRT as this forms the translational backbone of the study. IHC staining of the specimen will provide ‘proof of concept’ that EnAd is present in the tumour. Additional specimens will be fresh frozen for whole genome RNA sequencing focused on DNA damage repair pathways and immune signalling to understand mechanistic effects.

#### Dose limiting toxicity (DLT)

All patients who have received at least one dose of enadenotucirev will be evaluable for DLTs and are defined as any of the following occurring between the start of trial treatment until the Week 13 visit and by the principal investigator (PI) assessed as possibly, probably or definitely related to enadenotucirev or the interaction between enadenotucirev and radiotherapy and/or capecitabine. DLTs must be reported centrally within 24 h of the site becoming aware.

## Discussion

Attempts to improve standard of care radiotherapy for rectal cancer have failed. Additional chemotherapy, such as oxaliplatin or irinotecan, have not meaningfully improved outcomes to date, whilst concurrent biologic therapies (vascular endothelial growth factor (VEGF), epidermal growth factor receptor (EGFR), Poly(ADP-ribose) polymerase (PARP) inhibitors, etc.) have shown promising results at the expense of increased toxicity [39]. The reasons for failure are not purely biological however. A recent review of Phase 2 trials in rectal radiotherapy identified a number of issues including poor trial design, heterogeneous patient groups, lack of a validated efficacy endpoint as well as selection and reporting bias [40].

There is a logical case for the additive, or even synergistic, effect of oncolytic adenoviruses in combination with radiotherapy. The novelty in this trial is that, for the first time, we will be able to administer an oncolytic adenovirus systemically knowing it has very high selectivity for colorectal cells and negligible ability to infect normal tissues resulting in a very high hypothetical therapeutic index. Furthermore, the issues of improved radiosensitization in rectal cancer, mobilising an immune response and targeting micrometastatic disease can all be explored in the parallel translational program. It is uncommon to perform a phase I trial in a radically treated population who will receive curative surgery. But rectal cancer offers the unique ability to explore translational hypotheses because of the sequence in which standard of care clinical treatments are delivered, an opportunity increasingly availed of [41]. Patient and public involvement feedback has been very positive also.

CEDAR is methodologically novel and efficient by design as well as multicentre. Although it requires significant statistical support and engagement, our model will predict an optimal dose and schedule of administration to balance toxicity and efficacy. This should improve a subsequent Phase II study with preliminary estimates with which to power the chosen endpoint. The NAR score, a secondary endpoint, is a validated predictor of both OS [35] and DFS [42]. Rapid readout following surgery will allow for an early assessment of a very strong response signal even if the traditional pathological complete response remains at the expected 12–15%. These factors will attempt to overcome the previously described flaws which have hampered treatment intensification for rectal radiotherapy.

Two recent high profile consensus statements on novel drug radiotherapy combinations [4, 5] highlighted five key messages: 1) the potential of combinations to improve outcomes, 2) importance of communication between industry, academia, regulatory agencies and patient advocates, 3) intelligent trial design, 4) validated endpoints and 5) novel approaches including immune-oncology combined with radiotherapy should be prioritised. CEDAR is a bold new paradigm in the treatment of rectal cancer which addresses all of these key aims. The design and embedded translational program of work will hopefully result in rapid progress to wider clinical study, whilst underpinning the heretofore unanswered in-vivo mechanistic questions of enadenotucirev in rectal cancer.

## Data Availability

Additional data and materials may be requested from Professor Maria Hawkins.

## Abbreviations

TiTE-CRM: Time-to-Event Continual Reassessment Model
CEDAR: Chemoradiation with Enadenotucirev as a radiosensitiser in locally Advanced Rectal cancer
LARC: Locally advanced rectal cancer
CRT: Chemoradiotherapy
EnAd: Endadenotucirev
MTD: Maximum tolerated dose
CRC: Colorectal cancer
DDR: DNA damage response
NAR: Neoadjuvant rectal score
ICH: Immunohistochemistry
GTVp: Gross tumour volume of the primary disease
GTVn: Gross tumour volume of the radiologically involved lymph nodes
CTV: Clinical tumour volume
PTV: Planning target volume
DLT: Does limiting toxicity
EWOC: Escalation with overdose control
OS: Overall survival
DFS: Disease free survival
